# Microstructural controls of anticrack nucleation in highly porous brittle solids

**DOI:** 10.1038/s41598-020-67926-2

**Published:** 2020-07-24

**Authors:** Jonas Ritter, Henning Löwe, Johan Gaume

**Affiliations:** 1 Institute of Materials Simulation (WW8) and Central Institute for Scientific Computing (ZISC), Friedrich-Alexander-Universität Erlangen-Nürnberg (FAU), Erlangen, Germany; 2 WSL Institute for Snow and Avalanche Research SLF, Davos Dorf, Switzerland; 3 SLAB Snow and Avalanche Simulation Laboratory, EPFL Swiss Federal Institute of Technology, Lausanne, Switzerland

**Keywords:** Natural hazards, Solid Earth sciences, Materials science, Physics

## Abstract

Porous brittle solids have the ability to collapse and fail even under compressive stresses. In fracture mechanics, this singular behavior, often referred to as anticrack, demands for appropriate continuum models to predict the catastrophic failure. To identify universal controls of anticracks, we link the microstructure of a porous solid with its yield surface at the onset of plastic flow. We utilize an assembly method for porous structures, which allows to independently vary microstructural properties (density and coordination number) and perform discrete element simulations under mixed-mode (shear-compression) loading. In rescaled stress coordinates, the concurrent influence of the microstructural properties can be cast into a universal, ellipsoidal form of the yield surface that reveals an associative plastic flow rule, as a common feature of these materials. Our results constitute a constructive approach for continuum modeling of anticrack nucleation and propagation in highly porous brittle, engineering and geo-materials.

## Introduction

The compression of porous brittle materials can lead to localization of compaction or compacting shear bands^1–3^. This singular behavior, referred to as anticrack, originates from microstructural failure processes and has been observed in the compression of porous sandstone^4,5^, submarine landslides^6^, firnquakes^7^ or snow avalanches^8–10^. The latter is a particularly spectacular consequence of the nucleation of an anticrack under mixed mode loading: Snow as a highly porous material continuously changes its microstructure under thermodynamic forcing^11^. Under a high temperature gradient, a layer of small rounded crystals can turn into a weak layer of large faceted crystals^12^. Buried in a snowpack on a mountain slope, such a layer is always subjected to mixed-mode (shear-compressive) loading and the nucleation of an anticrack can lead to widespread anticrack propagation and dangerous slab avalanches^3,8^. To understand the generic mechanisms underlying these processes, irrespective of a particular material, it is necessary to decipher how the macroscopic behavior of mixed-mode anticrack nucleation originates from the microstructure.

**Figure 1:**
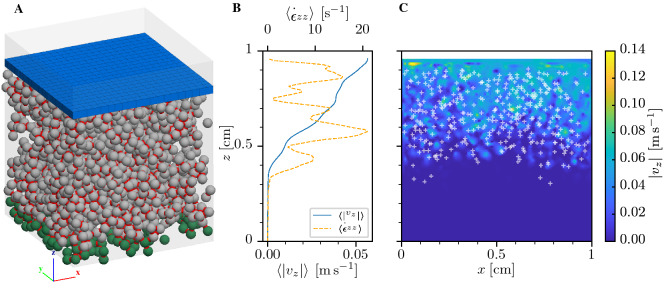
(**A**) Simulated system with fixed particles (green). Red necks represents bonds between particles. The loading clump is represented in blue. (**B**) Average vertical velocity $\langle |{v_z}|\rangle$ and average vertical strain rate $\langle \dot{\epsilon }_{zz}\rangle$ along the sample’s height. (**C**) Spatial distribution of the absolute value of the vertical velocity $|{v_z}|$ and (*x*, *z*) coordinates of broken bonds (white crosses) for a configuration with a volume fraction $\phi _0=0.3$, a coordination number $z_c=2.87$ and a deformation of 8%. Broken bonds are located within a compacted region in the upper $\sim$ 0.5 cm of the sample. Below this compacted zone, the sample remained mostly undisturbed.

From a continuum point of view, the nucleation of mixed-mode anticracks is controlled by the complex interplay between the yield surface, plastic flow rule and strain softening. It has been suggested that non-associated plasticity is necessary to reproduce anticracks in some porous sandstones^13^. In contrast, recent work on snow has demonstrated that anticrack nucleation and propagation in snow can be simulated using a continuum damage model with associated plasticity, if complemented by a modified strain softening law^3^. However, these question cannot be satisfactorily answered at the continuum level where involved assumptions cannot be traced back to and justified from the microstructure.

In the past decades, the extensive use of X-ray micro-computed tomography (XRCT) facilitated unprecedented insight into porous microstructures in all fields of material science^14^. Numerical simulations based on XRCT images allow to faithfully characterize and constrain the properties of the microstructural network (such as connectivity) on the mechanical behavior of different materials such as soils^15,16^, lunar soils^17^, snow^18–22^, rocks^23,24^ or concrete^25–27^. Simulations based on XRCT microstructure images are nowadays seen as complementary (numerical) experiments which can be repeatedly performed with different material properties, loading states and boundary conditions. This opens excellent opportunities in addition to (destructive) laboratory experiments which naturally lag behind in view of versatility and parameter variability. However, high resolution microstructure-based simulations still come with considerable computational requirements. Thus systematic large-strain, dynamic simulations for a comprehensive ensemble of different microstructures are still elaborate, which impedes the understanding of universal microstructural drivers of the complex mechanical behavior involved in anticracks.

A common approach to investigate microstructural controls on the mechanical behavior of porous solids are particle based methods such as DEM^28^. To access generic mechanisms relevant for a wide range of microstructural conditions, particle assemblies with volume fractions close to a dense packing are, however, not suitable. Rather a significant variation of the (mechanically) relevant parameters, i.e. volume fraction and the coordination number, are required to understand the controls of porosity and matrix connectivity on anticracks. As demonstrated by Gaume et al.^29^, Baxter’s sticky hard sphere (SHS) model^30^ can be conveniently utilized as a generic assembly method for DEM to independently prescribe volume fraction and coordination number, for very low porosity aggregates. In addition, a mapping of particle properties to continuous two-phase microstructures acquired by XRCT is principally feasible in the sense of a stochastic reconstruction, by matching two-point correlation functions^31^.

Here, we employ this methodology to systematically investigate a large ensemble of diverse microstructures with a wide range of coordination numbers and volume fractions within DEM under mixed-mode loading conditions (Fig. 1). From all simulations, we derive a single form of the continuum yield surface and the plastic flow rule relevant for anticrack nucleation. The results provide fundamental insight how the continuum mechanical behaviour of very loose and brittle solids is controlled by volume fraction and microstructural connectivity.

**Figure 2:**
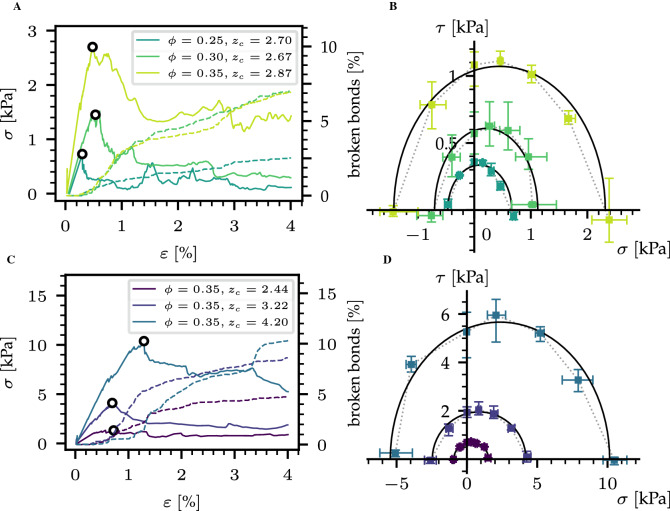
(**A**) Normal stress versus normal strain and percentage of broken bonds (dashed line) for uniaxial compression simulations for different volume fractions. (**B**) Yield surfaces obtained with mixed-mode loading simulations for different volume fractions. (**C**) Normal stress versus normal strain and percentage of broken bonds (dashed line) for uniaxial compression simulations for different coordination number values. (**D**) Yield surfaces obtained with mixed-mode loading simulations for different coordination number values.

## Results

### Mixed-mode anticrack nucleation

Heterogeneous compaction in brittle, highly porous solids is highlighted in uniaxial compression DEM simulations (Fig. 1C). The deformation occurs in a localized manner within a compacted region (illustrated by large vertical strain rates and distributed broken bonds in Fig. 1B,C) while the rest of the sample remains undisturbed. The generic behavior under uniaxial compression is shown in Fig. 2A,C for different volume fractions and coordination numbers. Stress–strain curves follow a quasi elastic brittle behavior i.e. (1) an almost linear elastic phase including non-cascading bond breaking events for low deformations up to (2) catastrophic failure followed by (3) strain softening. Increasing values of volume fractions and/or increasing values of coordination number lead to increasing elastic modulus and compressive strength. In addition, the increase in the broken bonds percentage is strongly correlated with the amount of softening after failure. After the strain-softening phase, samples collapse under the load leading to the localization of a compaction band^2^ (Fig. 1B) which is generally referred to as anticrack^4^. The residual stress after softening is the result of a competition between bond breaking and formation of new frictional contacts.

**Figure 3:**
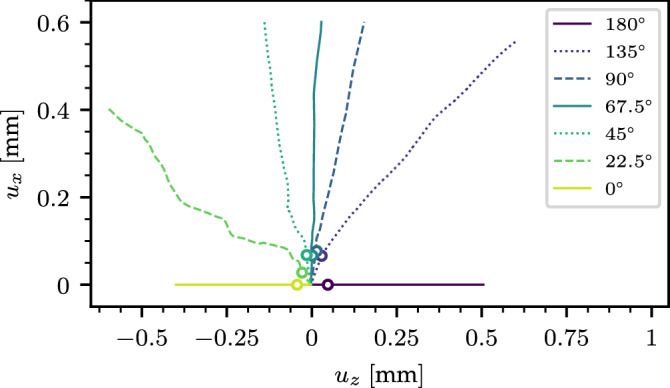
Volumetric response (tangential vs. horizontal displacement) for the configuration $\phi _{0} = 0.35$, $z_{c} = 2.00$ for different loading angles $\psi$.

The yield surface of the samples (Fig. 2B,D) was evaluated from mixed-mode loading simulations. All porous solid samples can fail under tension, shear, compression, and mixed-mode loading states leading to a closed yield surface. Tensile and shear strength are similar in magnitude, and both lower than the compressive strength. While the shape of the yield surface does not appear to be significantly affected by the initial microstructures, its size drastically increases with increasing volume fraction and coordination number, in line with the observation made for the compressive strength. Figure 3 shows the volumetric response of the samples for different loading angles. A transition between volume increase (expansion), and volume reduction (compaction) after failure is found for a loading angle of $\sim$ 67.5$^\circ$. For the latter angle, the vertical displacement of the clump $u_z$ remains almost constant during shearing. For larger compressive stresses, i.e. lower loading angles, fracture is followed by sample’s compaction (anticrack nucleation). In contrast, lower compressive stresses, i.e. larger loading angles lead to samples’ expansion after fracture (crack nucleation).

**Figure 4:**
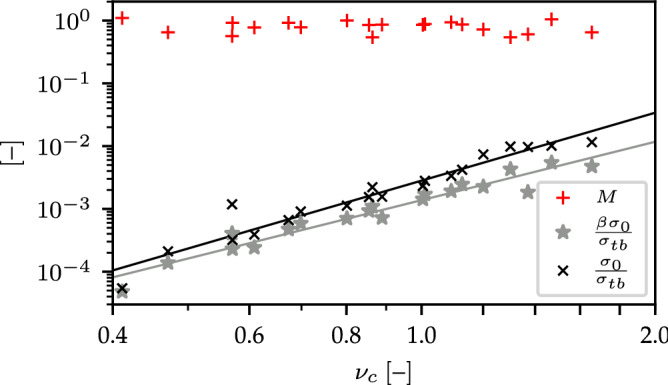
Compressive strength and tensile strength scaled by the bond tensile strength versus contact density as well as *M* versus contact density.

### Yield surface and plastic flow

Universal features were extracted by using a suitable continuum form of the yield surface, which could be re-scaled by inferring the dependence of the coefficients on volume fraction and coordination number. Given the elliptical shape of the samples’ yield surfaces, the Cohesive Cam Clay model developed by Gaume et al.^3^ was used to fit the simulation data in stress space of shear and normal stresses $\tau$ and $\sigma$, respectively. This yield surface was developed to simulate anticrack nucleation and propagation in snow and is defined here as:

1 $$\begin{aligned} \tau ^2(1+2\beta )+M^2(\sigma -\sigma _0)(\sigma +\beta \sigma _0)=0 \end{aligned}$$

where $\sigma _0$ is the compressive strength, $\beta \sigma _0$ is the tensile strength and *M* is the slope of the cohesionless critical state line^3^. The parameters $\sigma _0$, $\beta$ and *M* were evaluated using a least-square estimation. Examples of the resulting fitted ellipse are shown in Fig. 2 (black lines). Results show that the compressive stress and the tensile strength both scale with the contact density $\nu _c=z_c\phi$ of the samples according to a power law (Fig. 4):

2 $$\begin{aligned}&\frac{\sigma _0}{\sigma _{tb}}=h \nu ^{\kappa }_c \text {, }h=2.81\times 10^{-3}\text {, }\kappa =3.59 \end{aligned}$$

3 $$\begin{aligned}&\frac{\beta \sigma _0}{\sigma _{tb}}=k \nu ^{\lambda }_c \text {, }k=1.38\times 10^{-3}\text {, }\lambda =3.09. \end{aligned}$$

On the other hand, we did not find any trend for the parameter *M* which varies between 0.6 and 1 with an average of $\sim \,0.8$.

**Figure 5:**
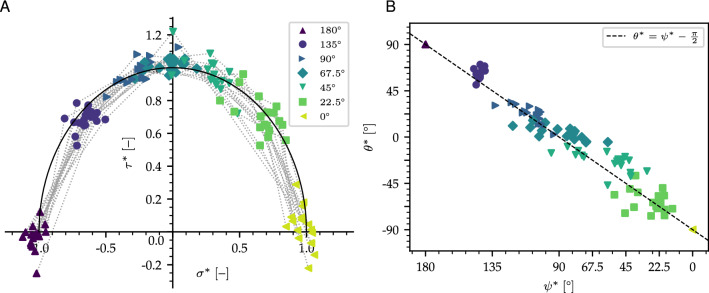
(**A**) Normalized failure envelopes for all configurations. (**B**) Related plastic flow angle versus loading angle in the normalized stress space.

Given the above scaling, it appears natural to search for a universal form of the yield surface parameterized based on contact density only. To this end, we define a transformed stress coordinates $\left( \sigma ^*,\tau ^*\right)$ in which the yield surface is a unit circle:

4 $$\begin{aligned} \tau *^2+\sigma *^2=1 \end{aligned}$$

To satisfy Eq. (1), $\sigma ^*$ and $\tau ^*$ are defined as

5 $$\begin{aligned} \sigma ^*= & {} \frac{2\sigma - (1-\beta )\sigma _0}{(1+\beta )\sigma _0} \end{aligned}$$

6 $$\begin{aligned} \tau ^*= & {} \frac{2\tau \sqrt{1+2\beta }}{M(1+\beta )\sigma _0} \end{aligned}$$

Using this normalization based on contact density only, all failure points collapse on a universal yield surface defined by Eq. (4) (Fig. 5A). Interestingly, the apex of the ellipse corresponds to a loading angle $\psi$ between 45$^\circ$ and 90$^\circ$ with a mean value $\sim$ 67.5$^\circ$. This angle corresponds to the transition between samples’ compaction and expansion. To evaluate the flow rule of the samples, we evaluate the plastic flow angle $\theta =\arctan \frac{u_z}{u_x}$ based on the simulations. To verify the assumption that the samples have an associative plastic flow rule, we relate $\theta$ to the derivative of the yield surface $\frac{\partial \tau }{\partial \sigma }$, which depends on the loading angle $\psi$. For the sake of universality, calculations are performed in the normalized stress space. Details of the calculations are presented in the Methods section.

Figure 5B shows the normalized plastic flow angle $\theta ^*$ as a function of the normalized loading angle $\psi ^*$. All data points appear to collapse on a single universal linear curve given by Eq. (16) which indicates that highly porous brittle solids follow an associative flow rule. Note that the loading angle $\psi ^{+/-}$ corresponding to a transition between expansion and compaction directly depends on the slope of the critical state line *M*, as well as the parameter $\beta$, which characterizes the cohesion of the material according to

7 $$\begin{aligned} \tan \psi ^{+/-}=\frac{M(1+\beta )}{(1-\beta )\sqrt{1+2\beta }} \end{aligned}$$

For a cohesionless material ($\beta =0$), $\psi ^{+/-}=\arctan M$ (critical state line) while for a porous solid with the same compressive and tensile strengths ($\beta =1$), $\psi ^{+/-}=\pi /2$ which is typical for metallic foams^32^.

## Discussion

An explanation as to why the yield surface is predominantly controlled by volume fraction and contact density is suggested by the fact that for arbitrary particle-based two-phase systems, the link between physical and structural properties is completely embodied in the hierarchy of $n-$point correlation functions^33^. The lowest point order ($n=1$) is only determined by the volume fraction as the widely accepted, most important parameter. In the next higher point order ($n=2$), the contact density $\nu _c$ controls the short-range expansion of the corresponding correlation function^34^. This implies that the contact density must be considered as the the second-most important parameter (beyond the volume fraction) in the combined expansion of the microstructure, with respect to point-order and spatial range. This naturally raises the question to which extent these two parameters can explain the macroscopic yield surface of highly porous brittle solids.

To this end, we considered a zoo of highly porous microstructures generated from the sticky hard sphere ensemble^30^ to independently control volume fraction and contact density. Within numerical uncertainties, both parameters are sufficient to quantify the yield surface and plastic flow rule of the porous solids. The yield surface of the samples collapse on a master curve (unit ellipse), after rescaling by the contact density. In addition, we have numerically shown that the plastic flow rule is associative. As a consequence, the volumetric response critically depends on the applied pressure. For large normal stresses ($\sigma >\frac{\sigma _0(1-\beta )}{2}$, i.e. on the right side of the apex of the ellipse), the plastic flow angle is negative leading to the volumetric collapse of the samples (plastic compaction). This process is referred to as a mixed-mode (shear-compression) anticrack. Together with previous results^29^ on the unique link between elastic modulus and contact density, our present results highlight the universal microstructural control of this quantity for the (pre-failure) mechanical behavior of highly porous brittle solids.

The proof of associativity presented here allows us to justify a strong assumption that was previously required to model anticrack nucleation leading to catastrophic slab avalanches in the recent work of Gaume et al.^3^. In this approach, a continuum model for dynamic anticrack propagation in snow has been developed based on critical state plasticity theory. These authors used a similar ellipsoidal (Modified Cam Clay) yield surface and made the assumption of an associative plastic flow rule to simulate the volume change during snow deformation. In addition to the proof of associativity, the present work allows us to evaluate the parameters of the model based on a single microstructural quantity: the contact density.

As aforementioned, the advantage of this approach is that parameters of the Sticky Hard Spheres model (volume fraction and coordination number) can be directly evaluated based on X-ray tomographic images by matching correlation functions^31^. While several recent numerical studies have analyzed the mechanical response of porous brittle solids like snow based on the real samples’ microstructures^18,19,21^, it has been recently shown that simplified structures made of spherical particles can be used to reproduce accurately important features of snow mechanics in the brittle range for different processes: failure initiation^35,36^, crack propagation^9,10^, snowflake fragmentation^37^, wind blowing snow^38^, snow granulation^39^ and avalanche impact pressures^40^. One the one hand, this simplification makes us loose important information about the microstructure. However, on the other hand, it allows us to significantly fasten the simulations and thus make detailed parameter sensitivity studies, which is not possible with highly detailed representations of the microstructure. In addition, the individual particle properties were chosen according to the ice mechanical behavior which constitute the solid matrix of snow. Yet, it was shown^29^ that the bulk elasticity and strength of the samples linearly scales with the particle elasticity and bond strength, respectively. This is reflected by Equations (2) and (3) in which the yield surface parameters are presented in a normalized manner, which allows to apply our results to other highly porous materials with different solid matrix properties. The presented DEM model is able to reproduce the nucleation and propagation of anticracks observed in porous layers of snow in the brittle range^3^. The formation of new cohesive bonds during the simulation would allow us in the future to reproduce the ductile-to-brittle transition in snow and thus the repetitive formation and reflection of compaction bands, as observed by Barraclough et al.^2^. Additionally, implementing a particle breakage criterion^41,42^ would enable us to simulate more complex types of localized deformation such as erratic and oscillatory compaction bands, observed in the compression of rice crispies^1^.

Evaluating the conditions for the onset of localization of compacting shear bands or anticrack nucleation in porous rocks is a great challenge^5,43^ and has implication for the understanding of deep earthquakes. Many associative plasticity models have been developed for porous rocks mechanics but localization is only possible with strain-softening, which occurs only under conditions of volume increase (dilation) according to classical critical state soil mechanics (CSM) models inspired by the behavior of granular materials. However, localized deformation was also reported under compressive stresses leading to compaction or compacting-shear bands^1–4,44–46^. Some researchers tried to overcome the inability of classical associative CSM models to reproduce anticracks or compaction bands through the development of non-associative flow rules. However, important discrepancies between experiments and model predictions have been found concerning localization features^13,43,47^. Wong et al.^47^ attributed differences between experimental observations and localization analysis to the “inadequacy of the non-associative constitutive model to capture the partitioning of several damage mechanisms, including the growth and coalescence of stress-induced microcracks and pore collapse”. The present analysis suggests that the plastic behavior of highly porous solids ($\phi <0.35$) is associative. The fact is, highly porous brittle solids undergo significant softening, even under large compressive stresses^1–3^. Gaume et al.^3,29^ suggested that the solid structure of porous solids under compression is actually under tension, which jeopardizes the continuum assumption. Hence, they proposed a modified hardening law based on the norm of the volumetric plastic strain, which leads to a shrinkage of the yield surface, even under compression until it corresponds to a point in the origin of the stress space, when cohesion is set to zero. The behavior under compression (on the cap of the yield surface) thus becomes similar to tensile mode I crack opening, which is refereed to as anticrack here (negative mode I). Here, simulations are performed for relatively low deformations ($<5$%) corresponding to the nucleation of the mixed-mode anticrack. For larger strain values, the yield surface changes significantly^3^. The value of $\beta$ decreases due to cohesion loss and $\sigma _0$ increases because of the creation of new frictional contacts. The decrease of $\beta$ induces a change in the value of the slope of the apparent critical state line (CSL). In the current formulation of the yield surface, *M* is the slope of the cohesionless CSL. Hence, the slope of the cohesive CSL which starts from the coordinate ($-\beta \sigma _0$, 0) is $M'=M/\sqrt{1+2\beta }$. Most porous geomaterials, including rocks and snow have values of $\beta$ between 0.1 and 0.5 typically^19,48,49^ leading to an increase of the apparent critical state line after failure of 10 to 40%. Hence, the difference observed between the plastic flow direction and the normality condition to the initial yield surface of highly porous solids is related to a sudden change of the shape of the yield surface induced by the post-peak softening.

Finally, we note that the choice of volume fractions for the simulations is motivated by the applicability to highly porous systems below the close packing density where the used Monte Carlo method of mono-disperse spheres^50^ may fail. At high volume fractions (close to the random close packing density, as obtained by Kun et al.^51^ using a different packing algorithm) the observed compaction band regime is generally complemented by a regime of predominant shear bands. It would be interesting to study such crossover behavior in the future using a microstructure assembly method that is able to cover the whole range of volume fractions.

## Methods

**Figure 6:**
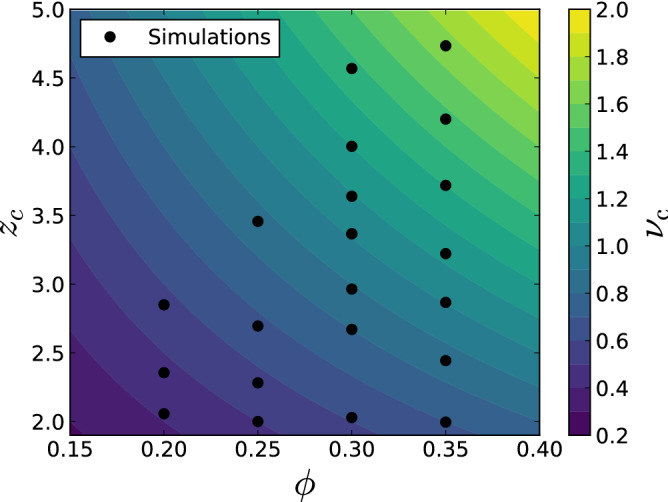
Values of coordination number $z_{c}$ and volume fraction $\phi$ used as input of the SHS simulations to generate the initial states of the DEM simulations.

### Initial states

The initial states of DEM simulations are generated using Baxter’s model of Sticky Hard Spheres (SHS)^30^. SHS allows us to generate a random assembly of percolating spheres of given volume fraction $\phi$ and coordination number $z_c$ (average number of contacts per sphere). Note that the SHS ensemble contains a percolation transition where the fraction of particles in the load-bearing backbone of the microstructures approaches zero. Naturally, using SHS state in the non-percolating phase is meaningless for their interpretation as a porous brittle solid. The details of the SHS model can be found in Gaume et al.^29^.

We used the same microstructures as in Gaume et al.^29^. They are generated through Monte-Carlo simulations based on the SHS model. Through this statistical approach it is possible to generate different realizations of a sample with the same initial volume fraction $\phi$ and initial average coordination number $z_{c}$ but random microstructures. For every combination of $\phi$ and $z_{c}$ (Fig. 6), three realizations were simulated.

### Discrete element simulations

We used PFC3D v5 by Itasca^52^ to simulate the mixed-mode loading of porous cohesive granular samples inside a cubic box of 1 cm side-length. The simulations are performed without gravity which allowed us to use a large particle density $\rho =10$,000 kg/m$^3$ to speed up the simulations.

Initial positions $(x_{p0},y_{p0},z_{p0})$ and particle radii *r* are obtained from Monte Carlo simulations. Simulations are performed with $N=2$,048 particles which was shown large enough to prevent size effects^29^.

All particles close to the bottom of the system ($z_{p0}<3r$) were fixed in translational and rotational movement (green particles in Fig. 1A). On the side, periodic boundary conditions were applied (transparent gray domain in Fig. 1A). At the top, a rigid clump was created to apply the mixed-mode loading.

Initial contacts between particles are bonded using the parallel bond model described in detail in Gaume et al.^29^. During the simulation, bonds can only break and new frictional cohesionless contacts can occur. Bonds have an elastic modulus of $E=1$ GPa and a normal-to-shear stiffness ratio $\kappa =3$. Bonds can break under shear and tension and have specific shear and tensile strength $\sigma _{tb}$ and $\tau _{sb}$, respectively. We chose $\sigma _{tb}=\tau _{sb}=1$ MPa. This leads to a bond failure strain of $\epsilon _{fb}=0.001$ of the order of that of ice.

### Loading and stress measure

Load-controlled simulations are performed by applying an increasing force $\vec {F}$ on the clump with different loading angles. The force is defined as $\vec {F}=F(\sin \psi \vec {x} - \cos \psi \vec {z})$ where $\psi$ is the loading angle. A loading rate $\dot{F}=0.05$ N/s was chosen sufficiently small to ensure quasi-static conditions. In addition, we verified that the inertial number^53^ during the simulation was sufficiently small ($I<10^{-4}$) to prevent the influence of inertial effects on the presented results. The shear and normal stresses $\tau$ and $\sigma$ were calculated at the bottom of the sample as

$$\begin{aligned} \frac{1}{A}\sum _{i\in N} f^i_x \quad \text {and} \quad \frac{1}{A}\sum _{i\in N} f^i_z, \end{aligned}$$

respectively. In the above expressions, *A* is the cross section of the sample, *N* represents the subset of particles fixed at the bottom of the sample, $f^i_x$ and $f^i_z$ are the resulting forces on particle *i* in the *x* and *z* directions, respectively.

### Failure identification

Failure was identified using a two-step criterion similar to that proposed by Mulak and Gaume^35^ based on the kinetic energy of the clump and the equivalent von Mises stress of the samples.

**Figure 7:**
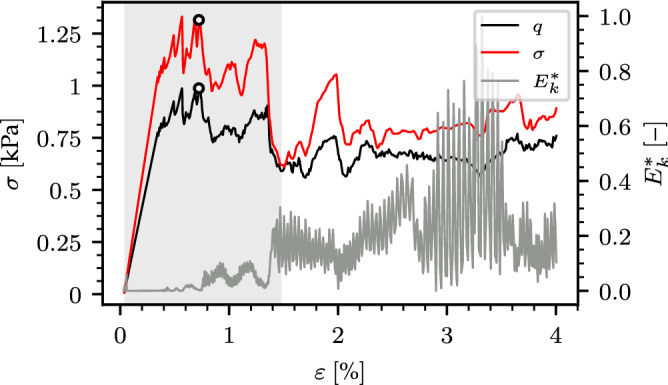
Identification of failure: normalized kinetic energy $E^*_k$ with the determined transparent gray search range and the equivalent von Mises stress *q* as well as normal stress $\sigma$. The identified failure is indicated by a black circle.

First we identify a strain range in which failure occurred (transparent gray range in Fig. 7). We define the normalized kinetic energy as:

8 $$\begin{aligned} E^*_k=\frac{E_k}{\max E_k}=\frac{\Vert {\varvec{v}}_c\Vert ^2}{\max \Vert {\varvec{v}}_c\Vert ^2} \end{aligned}$$

where ${\varvec{v}}_c$ is the clump velocity vector. After failure, $E^*_k$ significantly increases in all simulations. Hence, we define a strain range for failure by searching for $E^*_k<\zeta$. In a second step, we search for the maximum equivalent von Mises stress *q* within the previously defined strain range. The equivalent von Mises stress is defined as

9 $$\begin{aligned} q=\sqrt{\frac{3}{2}{\varvec{s}}\!:\!{\varvec{s}}} \end{aligned}$$

with ${\varvec{s}}$ as the deviatoric part of the stress tensor given by

10 $$\begin{aligned} {\varvec{s}} & = {\varvec{\sigma }}+p\mathbf {I} \end{aligned}$$

11 $$\begin{aligned} p= & {} -\frac{1}{3}\text {tr} {\varvec{\sigma }} \end{aligned}$$

where $\mathbf {I}$ is the identity matrix, *p* the pressure and, ${\varvec{\sigma }}$ is the Love homogenized stress tensor. The local maximum of *q* within the strain range defined above is defined as “failure point”. We verified, based on the stress–strain curves, that a value $\zeta =0.3$ led a very robust detection of failure. We also checked that the presented results remain valid for other failure criteria such as the second order work^54^ or a criterion based on the number of broken bonds.

### Plastic flow in the normalized stress space

The normalized loading direction is characterized by

12 $$\begin{aligned} \mu ^*=\tan \psi ^* \end{aligned}$$

with

13 $$\begin{aligned} \tau ^*=\mu ^*\sigma ^*. \end{aligned}$$

Combining Eqs. (4) and (12) yields

14 $$\begin{aligned} \sigma ^*=\frac{1}{\sqrt{1+\mu *^2}}. \end{aligned}$$

Hence, plugging $\mu ^*$ from Eq. (14) into Eq. (12) gives

15 $$\begin{aligned} \frac{\partial \tau ^*}{\partial \sigma ^*}=\frac{-\sigma ^*}{\sqrt{1-\sigma *^2}}=-\frac{1}{\mu ^*} \end{aligned}$$

For an associative plastic flow, the plastic flow angle $\theta ^*$ is given by

16 $$\begin{aligned} \theta ^*=\arctan \left( \frac{\partial \tau ^*}{\partial \sigma ^*}\right) =-\frac{\pi }{2}+\arctan \mu ^*=\psi ^*-\frac{\pi }{2}. \end{aligned}$$

Given the following relationship between the original and the normalized yield surfaces:

17 $$\begin{aligned} \frac{\partial \tau ^*}{\partial \sigma ^*}=\frac{\sqrt{1+2\beta }}{M}\frac{\partial \tau }{\partial \sigma } \end{aligned}$$

we obtain

18 $$\begin{aligned} \theta ^*=\arctan \left( \frac{\sqrt{1+2\beta }}{M}\frac{\partial \tau }{\partial \sigma }\right) =\arctan \left( \frac{\sqrt{1+2\beta }}{M}\frac{u_z}{u_x}\right) . \end{aligned}$$

The last piece of the puzzle consists in relating $\mu ^*$ to $\mu$ in Eq. (16). This is done by combining Eqs. (5), (6) and (13) and leads to

19 $$\begin{aligned} \mu ^*=\frac{\mu }{M}\sqrt{1+2\beta }\left( 1+\frac{1-\beta }{1+\beta }\sqrt{1+\mu *^2}\right) \end{aligned}$$

which can easily be solved for $\mu ^*$.

## References

[1] Guillard F, Golshan P, Shen L, Valdes JR, Einav I (2015). Dynamic patterns of compaction in brittle porous media. Nat. Phys..

[2] Barraclough TW (2017). Propagating compaction bands in confined compression of snow. Nat. Phys..

[3] Gaume J, Gast T, Teran J, van Herwijnen A, Jiang C (2018). Dynamic anticrack propagation in snow. Nat. Commun..

[4] Fletcher RC, Pollard DD (1981). Anticrack model for pressure solution surfaces. Geology.

[5] Sternlof KR, Karimi-Fard M, Pollard DD, Durlofsky LJ (2006). Flow and transport effects of compaction bands in sandstone at scales relevant to aquifer and reservoir management. Water Resour. Res..

[6] Locat, J., Leroueil, S., Locat, A. & Lee, H. Weak layers: their definition and classification from a geotechnical perspective. *Submarine Mass Movements and Their Consequences*** 3–12**, 10.1007/978-3-319-00972-8_1 (Springer International Publishing, 2014).

[7] Lough AC, Barcheck CG, Wiens DA, Nyblade A, Anandakrishnan S (2015). A previously unreported type of seismic source in the firn layer of the East Antarctic Ice Sheet. J. Geophys. Res. Earth Surf..

[8] Heierli J, Gumbsch P, Zaiser M (2008). Anticrack nucleation as triggering mechanism for snow slab avalanches. Science.

[9] Gaume J, van Herwijnen A, Chambon G, Birkeland KW, Schweizer J (2015). Modeling of crack propagation in weak snowpack layers using the discrete element method. The Cryosphere.

[10] Gaume J, van Herwijnen A, Chambon G, Wever N, Schweizer J (2017). Snow fracture in relation to slab avalanche release: critical state for the onset of crack propagation. The Cryosphere.

[11] Colbeck SC (1982). An overview of seasonal snow metamorphism. Rev. Geophys..

[12] Giddings JC, LaChapelle E (1962). The formation rate of depth hoar. J. Geophys. Res..

[13] Baud P, Vajdova V, Wong T-F (2006). Shear-enhanced compaction and strain localization: inelastic deformation and constitutive modeling of four porous sandstones. J. Geophys. Res. Solid Earth.

[14] Cnudde V, Boone MN (2013). High-resolution X-ray computed tomography in geosciences: a review of the current technology and applications. Earth Sci. Rev..

[15] Lim K-W, Kawamoto R, Andò E, Viggiani G, Andrade JE (2016). Multiscale characterization and modeling of granular materials through a computational mechanics avatar: a case study with experiment. Acta Geotech..

[16] Kawamoto R, Andò E, Viggiani G, Andrade JE (2018). All you need is shape: predicting shear banding in sand with LS-DEM. J. Mech. Phys. Solids.

[17] Matsushima T, Katagiri J, Uesugi K, Tsuchiyama A, Nakano T (2009). 3D shape characterization and image-based DEM simulation of the lunar soil simulant FJS-1. J. Aerosp. Eng..

[18] Hagenmuller P, Chambon G, Naaim M (2015). Microstructure-based modeling of snow mechanics: a discrete element approach. The Cryosphere.

[19] Srivastava PK, Chandel C, Mahajan P, Pankaj P (2016). Prediction of anisotropic elastic properties of snow from its microstructure. Cold Reg. Sci. Technol..

[20] Wautier A, Geindreau C, Flin F (2015). Linking snow microstructure to its macroscopic elastic stiffness tensor: a numerical homogenization method and its application to 3-D images from X-ray tomography. Geophys. Res. Lett..

[21] Köchle B, Schneebeli M (2014). Three-dimensional microstructure and numerical calculation of elastic properties of alpine snow with a focus on weak layers. J. Glaciol..

[22] Mede T, Chambon G, Hagenmuller P, Nicot F (2018). Snow failure modes under mixed loading. Geophys. Res. Lett..

[23] You Z, Adhikari S, Kutay ME (2009). Dynamic modulus simulation of the asphalt concrete using the X-ray computed tomography images. Mater. Struct..

[24] Shulakova V (2013). Computational elastic up-scaling of sandstone on the basis of X-ray micro-tomographic images. Geophys. Prospect..

[25] Skarżyński Ł, Nitka M, Tejchman J (2015). Modelling of concrete fracture at aggregate level using FEM and DEM based on X-ray CT images of internal structure. Eng. Fract. Mech..

[26] Ren W, Yang Z, Sharma R, Zhang C, Withers PJ (2015). Two-dimensional X-ray CT image based meso-scale fracture modelling of concrete. Eng. Fract. Mech..

[27] Huang Y, Yang Z, Ren W, Liu G, Zhang C (2015). 3D meso-scale fracture modelling and validation of concrete based on in-situ X-ray computed tomography images using damage plasticity model. Int. J. Solids Struct..

[28] Cundall PA, Strack ODL (1979). A discrete numerical model for granular assemblies. Géotechnique.

[29] Gaume J, Löwe H, Tan S, Tsang L (2017). Scaling laws for the mechanics of loose and cohesive granular materials based on Baxters sticky hard spheres. Phys. Rev. E..

[30] Baxter RJ (1968). Percus–Yevick equation for hard spheres with surface adhesion. J. Chem. Phys..

[31] Löwe H, Picard G (2015). Microwave scattering coefficient of snow in MEMLS and DMRT-ML revisited: the relevance of sticky hard spheres and tomography-based estimates of stickiness. The Cryosphere.

[32] Deshpande VS, Fleck NA (2000). Isotropic constitutive models for metallic foams. J. Mech. Phys. Solids.

[33] Torquato S., Stell G. (1982). Microstructure of two-phase random media. I. The n-point probability functions. J. Chem. Phys..

[34] Torquato S (2002). Random Heterogeneous Materials.

[35] Mulak D, Gaume J (2019). Numerical investigation of the mixed-mode failure of snow. Comput. Part. Mech..

[36] Bobillier G (2020). Micromechanical modeling of snow failure. The Cryosphere.

[37] Comola F, Kok JF, Gaume J, Paterna E, Lehning M (2017). Fragmentation of wind-blown snow crystals. Geophys. Res. Lett..

[38] Comola F, Gaume J, Kok JF, Lehning M (2019). Cohesion-induced enhancement of aeolian saltation. Geophys. Res. Lett..

[39] Steinkogler W, Gaume J, Löwe H, Sovilla B, Lehning M (2015). Granulation of snow: from tumbler experiments to discrete element simulations. J. Geophys. Res. Earth Surf..

[40] Kyburz ML, Sovilla B, Gaume J, Ancey C (2020). Decoupling the role of inertia, friction, and cohesion in dense granular avalanche pressure build-up on obstacles. J. Geophys. Res. Earth Surf..

[41] Zhang T, Zhang C (2019). Numerical simulation of particle breakage of granular assemblies in discrete element analyses. Adv. Civ. Eng..

[42] Zhou W (2020). Discrete element modeling of particle breakage considering different fragment replacement modes. Powder Technol..

[43] Bedford JD, Faulkner DR, Leclère H, Wheeler J (2018). High-resolution mapping of yield curve shape and evolution for porous rock: the effect of inelastic compaction on porous bassanite. J. Geophys. Res. Solid Earth.

[44] Mollema PN, Antonellini MA (1996). Compaction bands: a structural analog for anti-mode I cracks in aeolian sandstone. Tectonophysics.

[45] Olsson WA (1999). Theoretical and experimental investigation of compaction bands in porous rock. J. Geophys. Res. Solid Earth.

[46] Olsson WA, Holcomb DJ (2000). Compaction localization in porous rock. Geophys. Res. Lett..

[47] Wong T-F, Baud P, Klein E (2001). Localized failure modes in a compactant porous rock. Geophys. Res. Lett..

[48] Li L, Aubertin M (2003). A general relationship between porosity and uniaxial strength of engineering materials. Can. J. Civ. Eng..

[49] Reiweger I, Gaume J, Schweizer J (2015). A new mixed-mode failure criterion for weak snowpack layers. Geophys. Res. Lett..

[50] Ding KH, Tsang L, Shih SE (2001). Monte Carlo simulations of particle positions for densely packed multispecies sticky particles. Microw. Opt. Technol. Lett..

[51] Kun F, Varga I, Lennartz-Sassinek S, Main IG (2013). Approach to failure in porous granular materials under compression. Phys. Rev. E.

[52] Potyondy DO, Cundall PA (2004). A bonded-particle model for rock. Int. J. Rock Mech. Min. Sci..

[53] MiDi GDR (2004). On dense granular flows. Eu. Phys. J. E.

[54] Nicot F, Daouadji A, Laouafa F, Darve F (2011). Second-order work, kinetic energy and diffuse failure in granular materials. Granul. Matter.

